# Fabrication of a Zircon Microfiltration Membrane for Culture Medium Sterilization

**DOI:** 10.3390/membranes13040399

**Published:** 2023-03-31

**Authors:** Zineb Khebli, Ferhat Bouzerara, Nourddine Brihi, Alberto Figoli, Francesca Russo, Francesco Galiano, Sadek Chahredine

**Affiliations:** 1 Laboratory of Condensed Matter Physics and Nanomaterials, Jijel University, Jijel 18000, Algeria; 2 Institute on Membrane Technology, ITM-CNR, Via P. Bucci, Cubo 17/C, 87030 Rende, Italy; 3 Biotechnology, Environment and Health Laboratory, Jijel University, Jijel 18000, Algeria

**Keywords:** ceramic membrane, support, microfiltration, bacteria removal, zircon

## Abstract

Multilayer ceramic membranes to be used for bacteria removal by filtration were prepared from ceramic materials. They consist of a macro-porous carrier, an intermediate layer and a thin separation layer at the top. Tubular and flat disc supports were prepared from silica sand and calcite (natural raw materials), using extrusion and uniaxial pressing methods, respectively. Making use of the slip casting technique, the silica sand intermediate layer and the zircon top-layer were deposited on the supports, in this order. The particle size and the sintering temperature for each layer were optimized to achieve a suitable pore size for the deposition of the next layer. Morphology, microstructures, pore characteristics, strength and permeability were also studied. Filtration tests were conducted to optimize the permeation performance of the membrane. Experimental results show that the total porosity and average pore size of the porous ceramic supports sintered at different temperatures within the range (1150–1300 °C), and lie in the ranges of 44–52% and 5–30 μm, respectively. For the ZrSiO_4_ top-layer, after firing at 1190 °C, a typical average pore size of about 0.3 μm and a thickness of about 70 μm were measured, while water permeability is estimated to a value of 440 lh^−1^m^−2^bar^−1^. Finally, the optimized membranes were tested in the sterilization of a culture medium. Filtration results show the efficiency of the zircon-deposited membranes for bacteria removal; indeed, the growth medium was found to be free of all microorganisms.

## 1. Introduction

Membranes have important applications in the food [1], biotechnology, chemical, petrochemical [2,3] and pharmaceutical industries [4,5]. Recent studies have shown that membrane filtration is a viable option for removing contaminants from aqueous solutions. Without being exhaustive, membrane separation applications include the removal of oil from water [6], toxic heavy metal ions [7,8,9,10], and dissolved salts [11] and the separation of components from solutions such as proteins and macromolecules [12], dyes, bacteria, viruses [13,14], enzymes, antibodies, hormones, and blood proteins [15].

In various fields, such as the food, microbiology and pharmaceutical industries, it is becoming very important to sterilize solutions media to reduce the risk of contamination [16]. Membranes are a good tool with which to do that. An example application is the proposed case of the sterilization of plant tissue culture media (also known as growth media; this was the subject of a previous work [14]). Today, specific cell types derived from plants can be cultured in synthetic media. The latter is highly suitable for most of the contaminants to thrive and flourish in a short time; this is because the growth medium contains all the substances required for the growth of microorganisms such as bacteria [17,18,19,20]. The presence of these microbes in the medium could result in increased tissue culture decay [21]. For this reason, it is necessary to sterilize the nutrient media in order to protect them from any sources of contamination. To achieve this goal, several techniques have been developed. They include autoclaving, radiation (UV, microwaves and X-rays) and chemical methods [22,23,24], as well as ethylene oxide and plasma treatments. Autoclaving is the most common technique, because of its low cost and ease of use [24]. However, sterilization at high temperatures can lead to the degradation of organic substances and may result in undesirable reactions [25,26,27,28]. As an example, many proteins, and vitamins are thermo-labile and may decompose during autoclaving. As a sterilization technique, membrane filtration is potentially efficient at removing microbial contamination without altering the physicochemical properties [16] or affecting the functionalities of the culture medium components. Consequently, the preparation of membranes intended for this application has received increasing attention, especially those made of ceramics. Indeed, they are very effective for liquids filtration, as they exhibit a narrow pore size distribution, a high porosity, and a high permeability; they also have outstanding mechanical durability and great thermal and chemical stability. Furthermore, ceramic membranes can be cleaned even with harsh chemicals which can guarantee a longer service life [29]. Moreover, ceramic membranes are less susceptible to microbial attacks and biological degradation [30]. Therefore, developing membranes with the appropriate technical characteristics for the concentration or separation of microorganisms, such as spores and bacteria, is an emerging trend. Presently, the inorganic membranes for microfiltration (MF) are usually made of silica, alumina, zirconium or titanium. Studies in this field are widely reported in literature [31,32,33,34,35]. In this study, the peculiar properties of zircon were exploited for the development of MF membranes with a top layer made of zirconium silicate. It must be noted that little research has focused on the use of zircon in the fabrication of ceramic membranes. The ceramic filters are usually constituted of a thick support (2 mm) and one or multiple thin membranes. The thin top layer is responsible for separating components; the porous ceramic support provides the necessary mechanical strength to the membrane top layer to withstand the stress induced by the pressure difference applied over the entire membrane, offering, at the same time, a low resistance to the filtrate flow. The most common supports fabrication processes used for membrane systems include extrusion [36,37], tape casting [38], dry pressing, slip casting [39], and centrifugal casting [40,41,42,43]. Amongst them, slip casting, centrifugal casting, and dry pressing methods are widely used in laboratories, while the preferred method in industry is extrusion. Extrusion is a technological process for the production of ceramic tubes. It is also a very common method for ceramic support preparation. In addition, this method is rather inexpensive compared to others, and is therefore economically viable for tubes manufacture. In the extrusion process, a stiff paste is compacted and shaped by forcing it through a nozzle. In general, the manufacturing process of tubular ceramic supports using this method includes the following steps: (i) mixing various materials such as raw materials, organic additives and other extrusion aid materials to form a paste; (ii) passing the paste through an extruder to form a tubular support; (iii) drying and firing the samples.

There are several methods for preparing top layers depending on the application requirements, the desired membrane structure, and the specific materials. The most common manufacturing processes are slip casting, spin coating, dip coating or more sophisticated techniques such as a centrifugal process. However, there have been many studies on the preparation and characterization of membranes produced by the slip casting method [44,45,46]. A deflocculated slip is usually prepared through the mixing of powder, aqueous solution, and water. The deposition of the slip on the support is performed by the slip casting method [45]. In the case of the tubular membranes, the tube is closed at one end and filled with the solution. The coating is carried out by capillary suction. The thin layer thickness is determined by the capillary pressure and is dependent on the support porosity, the coating time, and the suspension viscosity [46]. After being dried at room temperature, the layer is sintered at an appropriate temperature. This method has the advantages of shaping complex geometries and also it allows the achievement of good quality membrane layers in terms of homogeneity and smooth surface, which are crucial properties for potential filtration applications.

In our investigation, composite ceramic membranes were prepared in three main steps: fabrication of supports manufactured from natural materials such as silica sand (SiO_2_) and (CaCO_3_); the realization of an intermediate layer made from silica powder; and finally, the preparation of a selective top layer made of zircon. Secondly, structural characteristics and the mechanical and chemical stability of the prepared membranes were examined, and their performance was assessed (in terms of water permeability). Finally, the separation capability of the prepared zircon ceramic membrane was evaluated using MF tests on a culture medium solution.

## 2. Materials and Methods

### 2.1. Materials

Zircon (ZrSiO_4_), natural silica sand (SiO_2_),and calcite (CaCO_3_) were used as starting materials for the fabrication of ceramic membranes. Silica sand (SS) and calcite (CC) raw materials originating from two sites in east Algeria were used for the preparation of the supports. CC has a double role—it helps reduce the sintering temperature and acts as a pore forming agent. The organic additive, methylcellulose (from Sigma-Aldrich 3050 Spruce Street, Saint Louis, MO 63103 USA), was used (4 wt.% of the ceramic powder) as a binder, in order to improve the rheological properties of the paste that makes the supports. Commercially available zirconium silicate (ZrSiO_4_) (purchased from Helmut Kreutz GmbH Company, Postfach 1242. Haiger, Germany) was used as a powder to prepare the membrane toplayer. Hydroxyethyl cellulose (from Merck Schuchardt OHG. Str 85662 Hohenbrunn Germany) was used as the organic binder in top layer preparation, helping with tuning the sol viscosity and protecting the thin layer from cracking.

A culture medium was prepared for the validation of the sterilization process. It was basically an aqueous solution where all the needed nutrients had been added; it contained simple sugar as a carbon and energy source, various mineral salts (macro-elements, microelements), and growth factors (purified amino acids, vitamins, and pyrimidines).

### 2.2. Production of the Supports

Usually, ceramic MF membranes are obtained in three stages. First, the tubular or flat discs porous ceramic supports are prepared, followed by the application of an inter-layer at the second stage, concluding with the deposition of the MF membrane in the third stage.

The supports samples were prepared as follows: the first step was the preparation of a paste by mixing 75 g of SS as a major raw material with 25 g of CC powders and 4 g of organic additive (methyl cellulose) as a binder. Water was added to obtain a plastic paste with a good homogeneity and to facilitate the shaping, which was the next step in the sample formation process. In the present work, we used the following shapes:

- Tubular and flat rectangular supports were made by pressing the paste in a die, using a hydraulic press. The extruded tubes were placed on rotating rollers to cause the support to rotate so that they dried up and stayed straight. Tubular supports had the following dimensions: 6 mm (I.D.) and 10 mm (O.D.) for the inner and outer diameters, respectively, while the length of the supports was chosen according to our needs. They were used to study membrane features.
- Rectangular samples (with 40 × 8 × 6 mm) were prepared using the extrusion technique. These samples were used to estimate mechanical properties.
- Flat disc samples (with a diameter of 50 mm and a thickness of 2 mm) were prepared by hydrostatic pressure. They were used in the filtration tests.

**Figure 1:**
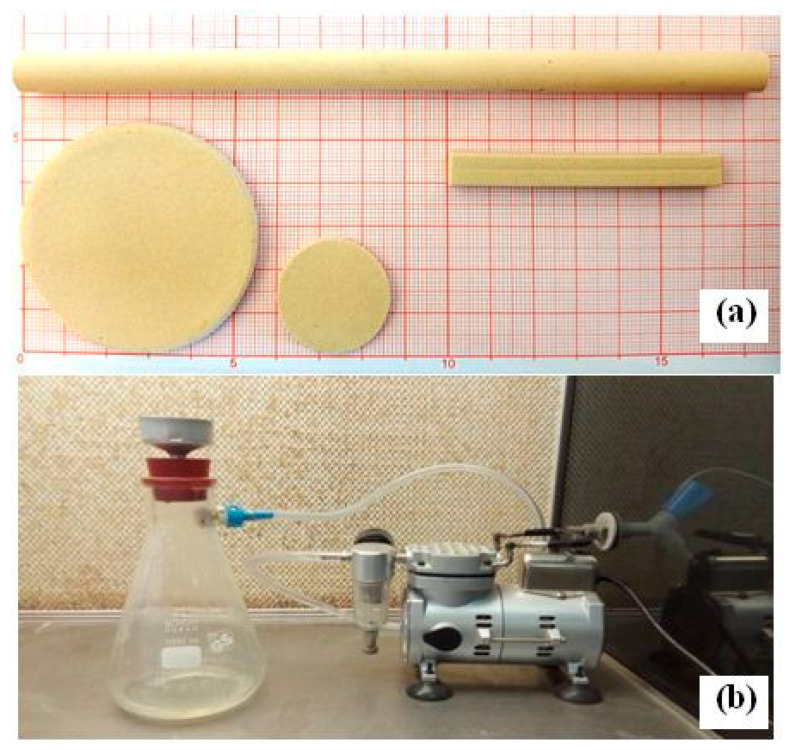
(**a**) A photograph of prepared samples: tube, flat disc, and flat rectangular. (**b**)A photograph of the experimental installation used in frontal MF.

Finally, after drying, the samples were fired at different temperatures in the range (1150–1300 °C) for 1 h, with a heating rate of about 5 °C/min, from 25 °C to T (°C). A picture of the obtained samples is presented as Figure 1a.

### 2.3. Membranes Preparation

The slip casting method was selected in order to obtain a porous ceramic membrane with a smooth surface and a uniform pore-size distribution. This technology includes preparation of the slip, casting, drying, and sintering.

An inter-layer was applied using a deflocculated suspension. The preparation process consisted of the following steps:

- Crushing of the powder and calibration at 40 μm by sieving;
- Dispersing the mineral powder (20 g) in distilled water (50 mL);
- Adding an aqueous solution of hydroxyethyl cellulose (30 g);
- Homogenizing by magnetic stirring followed by the deposition on a silica layer by using the slip casting method (the time of deposition is about 5 min);
- Drying followed by sintering at 1200 °C for 1 h at a heating rate of 5 °C/min.

The slip for the top layer was obtained by mixing 20 wt.% zircon powder (particle size <5 μm), 30 wt.% aqueous solution of hydroxyethyl cellulose, and 50 wt.% of distilled water (DW). Afterwards, it was deposited on a silica layer using the slip casting method. The time of deposition was about 5 min. Then, after drying at room temperature, the membrane was sintered at 1190 °C for 1 h. This temperature was selected because it resulted in good characteristics and good adhesion between support and membrane.

### 2.4. Culture Medium Preparation and Sterilization

In order to study the ability of the prepared membranes to perform the removal of bacterial cells, a Murashige and Skoog medium (MS), i.e., a laboratory plant tissue culture medium, was used. It consists of macronutrients (nitrogen, phosphorus, potassium, calcium, magnesium and sulfur), micronutrients (iron, manganese, zinc, boron, copper, cobalt and molybdenum) and also vitamins (glycine, thiamine (vitamin B1), nicotinic acid (also known as niacin or vitamin B3), pyridoxine (vitamin B6), myo-inositol, and a carbon resource (sucrose).

A standard medium solution was prepared, according to the method described in [14], whereby 200 mL of mineral solution was sterilized at a temperature of 121 °C for 20 min. Then, part of the organic non-sterile medium was filtered through the MF membrane (Figure 1b). The remaining part was sterilized by autoclave and poured into a flask containing the above sterilized mineral medium. It was then heated and solidified with agar. The flasks were tightly closed to prevent any contamination by microorganisms. In addition, flasks containing the same volume (100 mL) of sterilized mineral solution were supplemented with an organic non-sterilized medium (without the use of membrane or autoclave), to be used as a control test. After that, the flasks were placed in an incubator at 25 °C for 30 days. Visual checks on any variation (color, form) on the surface of the sterilized and non-sterilized media were made on a daily basis.

### 2.5. Characterization Techniques

Various characterization techniques were used to study the properties of the prepared membranes. Chemical analysis was carried out by means of X-ray fluorescence spectrometry (Zetium-Malvern Panalytical, Great Malvern, UK). Particle size distributions of raw materials were obtained using a laser diffraction particle size analyzer (HORIBA-LA-960, HORIBA, Kyoto, Japan). Thermal analysis (TGA/DSC) was performed using an SDT Q600 TA instrument, from 30 to 1300 °C at a heating rate of 10 °C/min in an air atmosphere.

The membranes were characterized by studying both their structure and functionality. Open porosity, average pore size or diameter (APS), and pore size distribution (PSD) were obtained using a mercury intrusion porosimetry technique (Micromeritics, Model Autopore 9220) for specimens sintered at different temperatures. The pore size was also evaluated by using the wet-up/dry-up method with a capillary flow porometer (POROLUXTM 1000 Porometer, IB-FT GmbH, 12277 Berlin, Germany). Structural properties of the samples were analyzed by physical adsorption of N_2_ at 77K using a Micromeritics ASAP 2460 apparatus. Before each adsorption measurement, all samples were dried under vacuum at 423 K for 12 h. The specific surface area (SSA) was calculated according to the Brunauer–Emmett–Teller (BET) method within a relative pressure range of 0.05–0.3.

The flexural strength of sintered samples was measured by mechanical experiments consisting of the three-point bending test. The flexural strength presented in this work was the average value resulting from five different samples. Phase analysis was conducted by X-ray diffraction (XRD) via a diffractometer (BRUKER-AXS-D8) with Cu_Kα_ radiation. Microstructure and morphology were examined using scanning electron microscopy (SEM, TESCAN VEGA3).

## 3. Results and Discussion

### 3.1. Raw Materials Characterization

**Figure 2:**
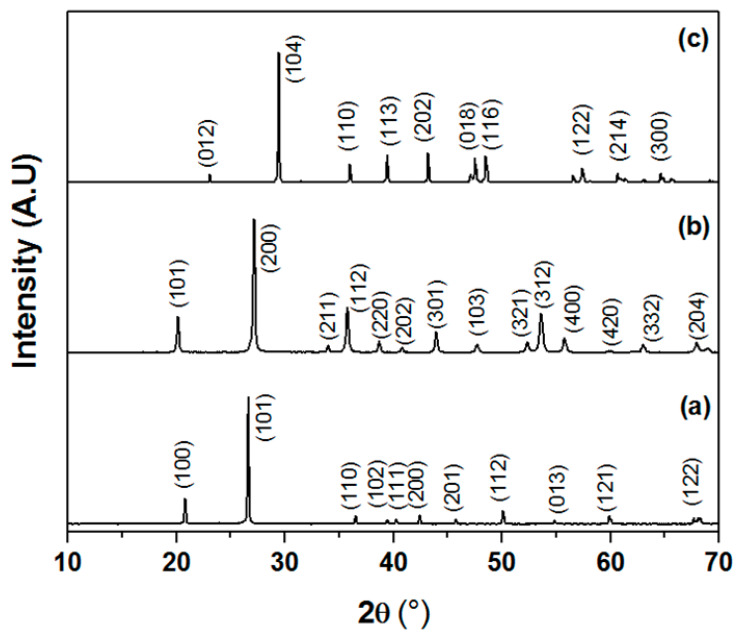
XRD spectra of raw materials, (a) SS, (b) zirconium silicate powders, and (c) CC powders, respectively.

**Figure 3:**
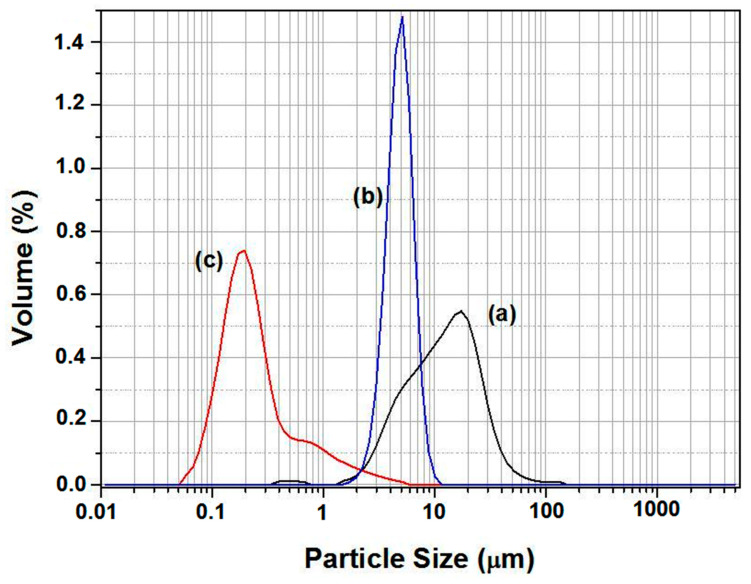
Particle size distribution for SS (a), CC powder sand (b), and zirconium silicate powders (c).

Chemical compositions of SS, CC and zircon were obtained by X-ray fluorescence as shown in Table 1. SS is mainly composed of silica at around 85 wt.%, 6 wt.% Al_2_O_3_, 2 wt.% CaO, and some impurities. The quantitative analysis of calcium carbonates (CaCO_3_) revealed that the purity of this material was about 99%. The chemical composition of zircon powder consists of SiO_2_ and ZrO_2_ as the major components. However, traces of Fe_2_O_3_ and TiO_2_ were also detected. Figure 2 presents the XRD spectra of SS, CC, and zircon. It shows that they are crystallized and that all peaks in the XRD patterns are, indeed, related to these raw materials. The XRD pattern of the SS confirms that quartz is the main crystalline mineral in this powder. The main phases detected in the zircon sample were quartz and zircon oxide (ZrO_2_). The XRD pattern of the calcium carbonate powder shows that only CaCO_3_ is present. The particle size distribution of used raw materials, measured by laser scattering technique, is provided in Figure 3. It shows the following particles size ranges: SS 2–60 μm; CC 1–10 μm; and zircon 0.1–5 μm. The estimated average particle size is of the order of about 14 µm, 5 µm, and 0.6 µm for SS, CC, and zircon, respectively. In additions, it is noticeable that SS powder presents an average grain size larger than that of zirconium silicate or CC.

**Figure 4:**
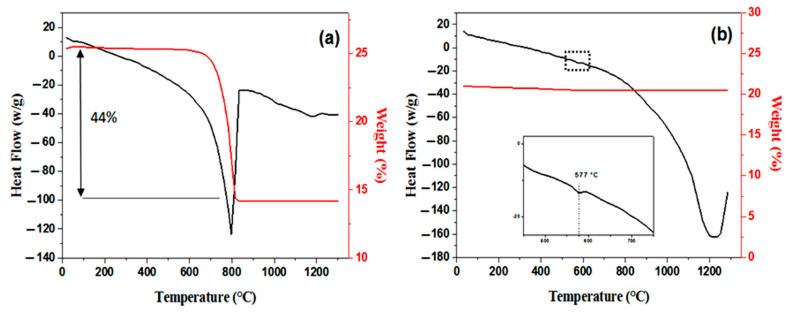
Thermal analysis curves (DSC and TGA) for (**a**) CC powders and (**b**) SS.

Figure 4 shows the TGA and DSC results for CC and SS; the samples were heated at 10 °C/min, from room temperature to 1300 °C, in an air atmosphere. The DSC curve of CC powder presents a broad endothermic peak in the temperature range of 600 to 850 °C, caused by the decarbonation of CC—CaCO_3_ transforming to CaO and CO_2_. The TGA curve shows that the endothermic process is accompanied by a decrease in mass. Indeed, it decreased by about 44% during this process. This is the case mostly above 600 °C and continues until about 850 °C.

The thermal analysis curves for SS are shown in Figure 4b. The DSC curve presents a small endothermic peak at about 577 °C, indicating some structural rearrangement at this temperature; the α-quartz, which has a trigonal symmetry, turns into hexagonal β-quartz [47,48]. The TGA curve slightly decreased (1% in mass) up to 450 °C.

### 3.2. Supports Characterizations

**Figure 5:**
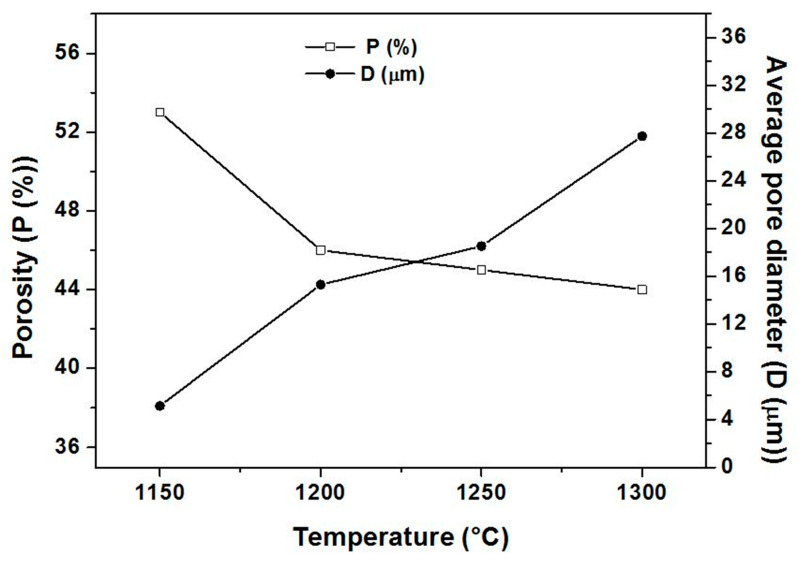
Variation of porosity ratio (P) and average pore diameter (D) as a function of the sintering temperature.

In this investigation, tubular supports, prepared using an extrusion technique, were used. The basic properties such as pore size, porosity, permeability and lifetime are crucial for membranes applications. To enhance membrane support performance, the effect of calcination temperatures on the properties of porous membranes supports was examined for a range of temperatures from 1150 to 1300 °C. Figure 5 shows the porosity ratio and the average pore size of ceramic supports as a function of the sintering temperature. As can be seen, the APS increases with the temperature increase. The APS of supports sintered at 1300 °C (27.7 µm) is five and a half times higher than that of specimens sintered at 1150 °C (5.1 µm). The increase in average pore diameter may be attributed to pores coalescence. In addition, experimental results (Figure 5) show that the porosity decreases with the increasing temperature. When the firing temperature was increased from 1150 °C to 1200 °C, the open porosity significantly decreased from 53% to 46% above 1200 °C, before it stabilized at a steady level. The sample calcined at 1150 °C exhibits the highest total porosity of 53%, while the lowest total porosity of 44% was observed for the sample calcined at 1300 °C. It can also be observed that the porosity reduction in the range 1150–1200 °C is more important than for 1200 to 1300 °C. However, the range of sintering temperatures from 1200 °C to 1300 °C has no effect on the variation of porosity percentage; this is attributed to the significant increase in pore size. Big pores are, in fact, more stable.

**Figure 6:**
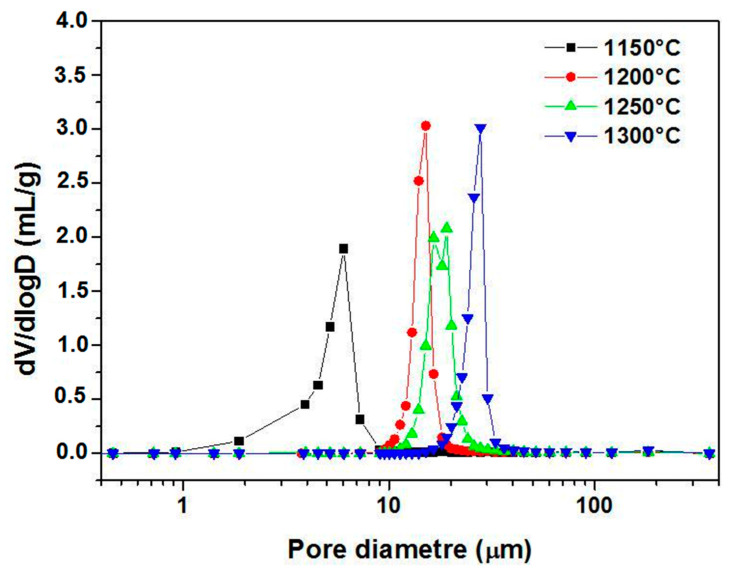
Pore size distribution for supports sintered at different temperatures for 1 h.

**Figure 7:**
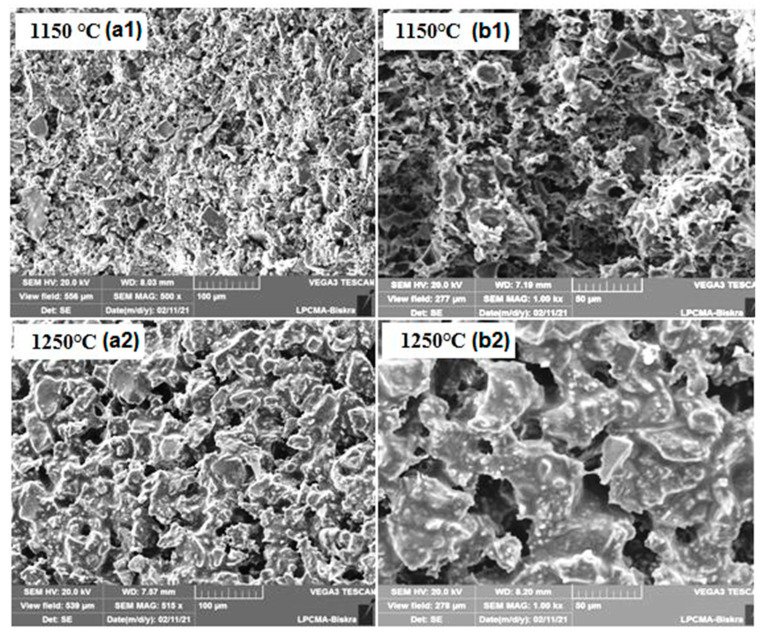
SEM micrographs of membrane supports sintered for 1 h at 1150 and 1250 °C, (**a1,a2**) surface and (**b1,b2**) cross-section.

**Figure 8:**
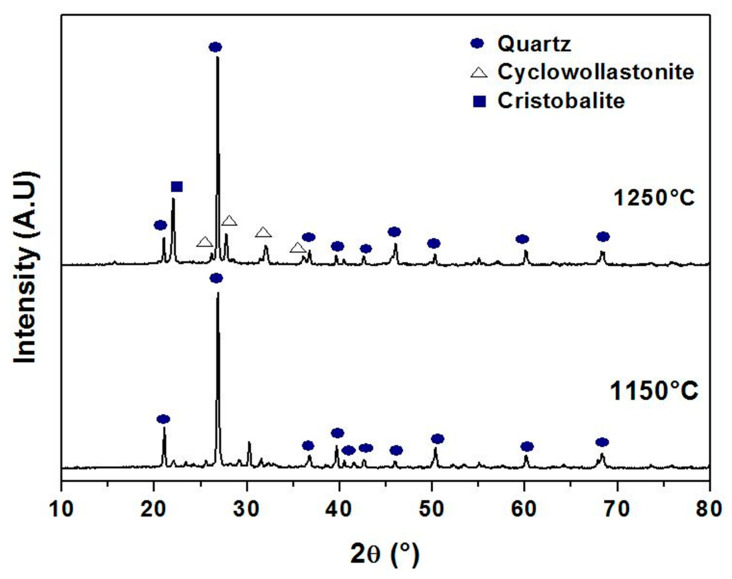
XRD spectra of support samples sintered at 1150 °C and 1250 °C for 1 h.

Figure 6 shows the pore size distributions (PSD) curves of samples fired at different temperatures. It was possible to affirm from the monomodal distributions patterns that most of the pores are in the micro-size. Figure 6 suggests that pores in samples fired at 1150 °C are between 1 and 10 μm. Nevertheless, while for the specimens sintered at 1200 and 1250 °C the majority of the pores have a diameter smaller than 30 μm (ranging from 10 to 30 μm), for the specimens sintered at 1300 °C, the majority of the pores show a diameter smaller than 40 μm (ranging from 20 to 40 μm). Furthermore, the average pore diameters are 5.1, 15.3, 18.5, and 27.7 μm corresponding to the temperatures 1150, 1200, 1250, and 1300 °C, respectively. As can be seen in Figure 6, when the sintering temperature rises from 1150 to 1300 °C, the PSD curve moves towards larger pore sizes, while the volume percentage of pores smaller than 10 μm decreases. This may be attributed to the growth of large pores at the expense of small pores. Furthermore, it can be noticed from the SEM image in Figure 7 that the pores in the ceramic supports are micro-sized and randomly shaped. The surface of membrane supports has a relatively uniform pore size distribution, and some large pores are also observed. Furthermore, there is an increase in the size of pores as the temperature increases to 1250 °C; this is consistent with the pore size distribution shown in Figure 6. XRD patterns of supports sintered at 1150 °C and 1250 °C for 1 h are illustrated in Figure 8. The main phase identified in the membrane supports sintered at 1150 °C is quartz (α-SiO_2_). However, after sintering at 1250 °C, two new phases (cyclowollastonite CaSiO_3_ and cristobalite (SiO_2_)) are formed; this happens when the quartz phase content decreases. The appearance of new lines and the change in intensity of some lines for samples fired at 1250 °C suggests that a chemical reaction between quartz and CC occurred, leading to the formation of cyclowollastonite.

**Figure 9:**
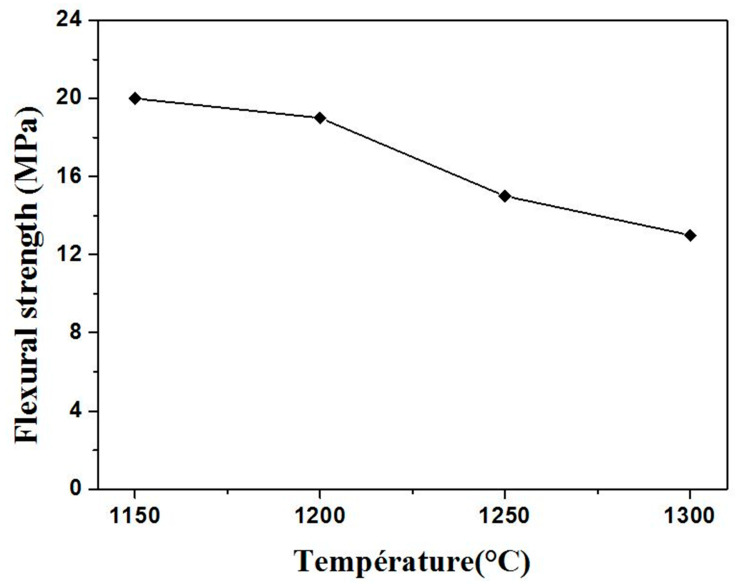
Variation of flexural strength as a function of the sintering temperature.

The flexural strength values were taken as the average of the measurements on five rectangular shape samples, using the bending three points test. Results for samples sintered at different temperatures are shown in Figure 9. There is a decrease in flexural strength when the sintering temperature is increased. As examples, for a sintering temperature of 1150 °C and 1 h heating, a flexural strength of 20 MPa, a porosity ratio ≈ 53%, and an APS ≈ 5 µm were found. In the case of an increased temperature to 1300 °C, the following values were found: flexural strength of 12 MPA, porosity ratio of 44%, and APS of 27 µm. When the sintering temperature is raised from 1150 to 1300 °C, the supports exhibit a slight increment in the bulk density and a sharp increase in pore size. Increasing the sintering temperature of samples caused a decrease in the porosity, paradoxically; it has also been associated with a decrease in the flexural strength of the samples, in fact, the mechanical strength of ceramics is known to rise when porosity decreases. However, samples show significant differences in pore size values. The presence of large pores leads to a decrease in the mechanical strength of ceramics resulting from the link between the flexural strength and the APS, which is sintering temperature-dependent. A comparison between supports sintered at 1200, 1250, and 1300 °C reveals superior values of flexural strength for supports at 1200 °C, but almost similar values of porosity ratio.

**Figure 10:**
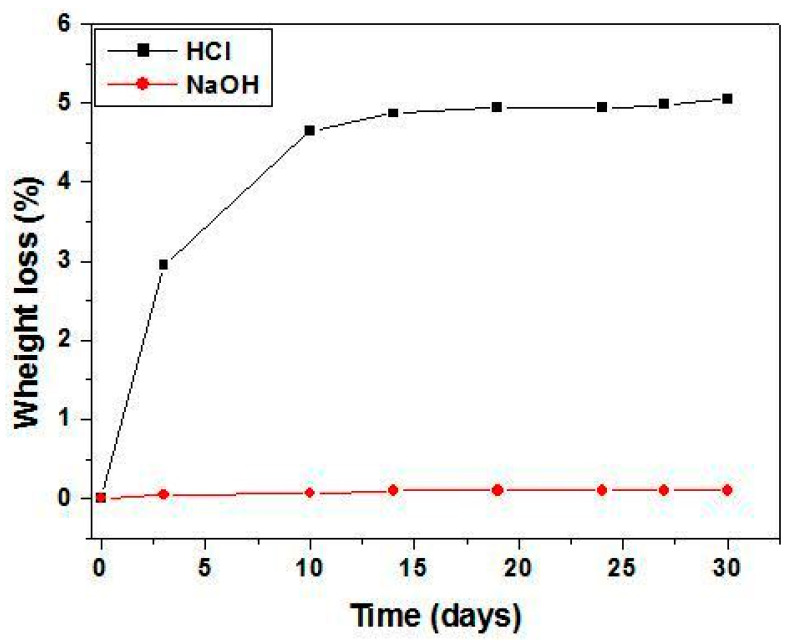
Weight loss of supports sintered at 1250 °C in the basic (NaOH) and acidic (HCl) solutions as a function of time.

Resistance to corrosion prolongs the lifetime of the supports. In order to assess this property, mass loss measurements were carried out. These were performed after having immersed the first sample into a basic solution of NaOH (pH ≈ 11.90), while for the second sample, an acidic HCl (pH ≈ 2.0) solution was used at a room temperature. Measurements were made as a function of immersion duration up to a maximum of 30 days. Figure 10 shows the results for these specimens, which were sintered at 1250 °C. It is possible to notice that the first support presents a high chemical resistance in basic media since its mass loss was much lower (around 0.1 wt.%) than for those subjected to acidic solution. On the other hand, the mass loss in the second case rose to a value of ≈4.9 wt.% after 10 days of immersion and remained stable at this value. Quality wise, this can be considered acceptable. Therefore, it is possible to conclude that the developed supports showed a satisfactory resistance to corrosion regardless of the acidic or basic nature of the medium.

**Figure 11:**
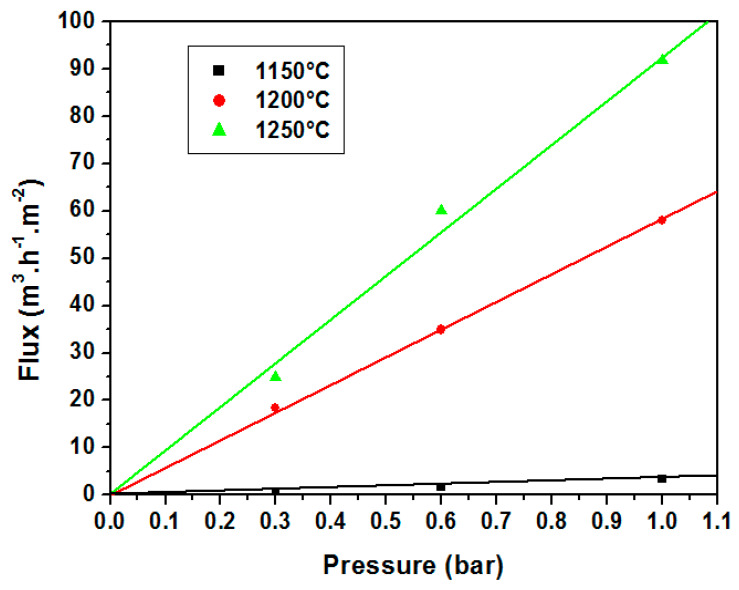
Water flux variation versus pressure for supports sintered at 1150, 1200, and 1250 °C.

After structural and mechanical characterization, permeability measurements are also of great importance since porous supports should have low resistance during filtration. Tangential water filtration experiments were performed using a home-made pilot plant, for the evaluation of their performance in terms of permeability. Figure 11 demonstrates the water flux through the supports sintered at 1150, 1200, and 1250 °C, as a function of the applied pressure. As can be seen from Figure 11, the flux increases linearly with the applied pressure difference (from 0.3 to 1 bar) for all samples; the average permeability was about 4, 56, and 95 (m^3^/(H·m^2^·bar)) for supports sintered at 1150, 1200, and 1250 °C, respectively. This high permeability is a very important property for the membrane supports performance. Furthermore, it is observed (Figure 11) that permeability increases with the firing temperature. Clearly, for the supports sintered at 1250 °C, the flux is relatively high compared to supports sintered at 1150 °C and 1200 °C. By looking at the trend of permeability, taking into consideration Figure 5 and Figure 7, it is clear that the increase in permeability with the firing temperature is related to changes in the microstructure and corresponds to an increase in pore size. This is because the flux (and hence permeability) is proportional to the open porosity and APS squared as can be shown by the Hagen–Poiseuille equation [49,50]. For practical applications, both water flux and strength should be as high as possible. On the basis of the obtained results, the best sintering temperature for a porous ceramic support was found to be about 1250 °C.

### 3.3. Membranes Characterization

The top layer is closely related to its support [11] and the quality of the support is of crucial importance to the integrity of the membrane layers that are applied on the top. The supports sintered at 1250 °C were selected as substrates. Because the surface of these supports has a relatively uniform pore size distribution (Figure 6) with a relatively large APS (18.5 μm), the interlayer was found to be an easy way to improve the surface roughness of the ceramic membrane supports and, thus, gradually reduce the pore size. The intermediate layer was made from SS (SiO_2_) powder (the same powder used for support elaboration) sintered at 1200 °C.The role of this layer is to minimize the surface defects and enhance surface roughness, as mentioned above. The outer layer was made of zircon.

**Figure 12:**
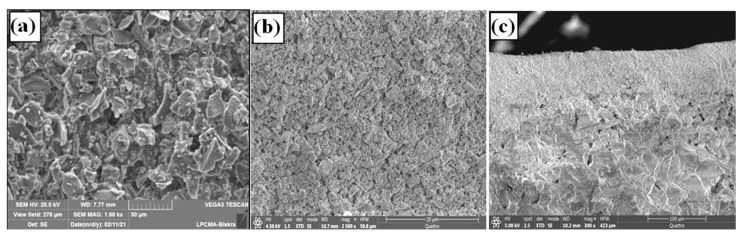
SEM micrographs; (**a**) surface of intermediate layer, (**b**) surface of a top layer (**c**), cross-section of the multilayer system.

The sintered membranes were examined by SEM and mercury porosimetry. Figure 12 shows the microstructures of the top layer and support on SEM micrographs. It can be seen that the intermediate and top layers present a smooth surface and a uniform structure. Moreover, cracks and large pores could not be observed on the surface of the interlayer, indicating that the surface quality of supports has improved. The membrane cross-section (Figure 12c) shows that the zircon top layer and the support constitute an asymmetrical structure. The zircon layer is dense, with a thickness of about 70 μm that can be controllable by adjusting the deposition time (the coating time) and the particle concentration in the slip.

**Figure 13:**
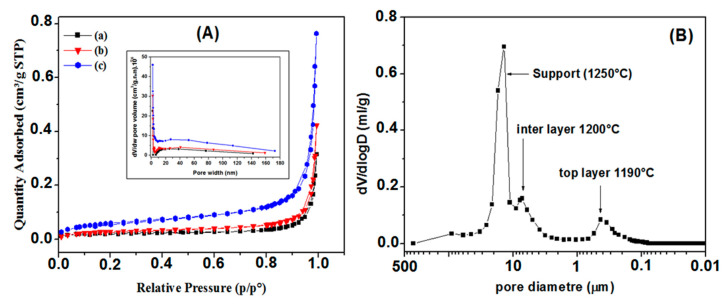
(**A**) Isotherms from nitrogen sorption measurements for: (a) support (1250°), (b) support + interlayer, (c) support + interlayer + top layer. (**B**) Pore size distribution for multilayer system.

Figure 13 presents the analysis results obtained through the two methods used to characterize the textural features of the membranes. Figure 13A shows the nitrogen adsorption/desorption isotherms, acquired at 77K, of the studied samples. All the samples show Type II isotherms with a Type H3 hysteresis loop, similar to those of powders and aggregates. According to the IUPAC classification [51], these samples are classified as mesoporous materials. The adsorption behavior in mesopores is determined by the adsorbent–adsorptive and adsorptive–adsorptive interactions. This leads to the occurrence of multilayer adsorption and capillary condensation, which are accompanied by a hysteresis curve whose shape is related to the texture of the adsorbent. H3 hysteresis is the result of inter-particle capillary condensation. From the nitrogen adsorption isotherms, it is evident that sample (c) has a relatively compact structure. Indeed, the specific surface area (SSA) increases with the number of layers. The resulting SSA measurements are 0.087, 0.113 and 0.222 m^2^g^−1^, corresponding to the samples (support), (support + interlayer), and (support + interlayer + top layer), respectively. The same behavior is observed for the total pore volume (V_T_). The smallest value, V_T_ = 47 × 10^−5^ cm^3^·g^−1^, corresponds to sample (a), the median value, V_T_ = 65 × 10^−5^ cm^3^·g^−1^, corresponds to sample (b), while the maximum value, 117 × 10^−5^ cm^3^·g^−1^, was obtained for sample (c). Figure 13B presents the pore size distributions of the as-prepared membrane. From this figure, the APS for the SS inter-layer and zircon top layer were estimated as 6 μm and 0.3 μm, respectively. The pore size distribution of the top layer is narrow, ranging from 0.1 to 0.6 μm; it is a single (mono) distribution modal, confirming that the membrane has a uniform pore size distribution and an APS equal to 0.3 μm (Figure 13B). This APS suggests that this kind of membrane can be utilized in MF applications.

**Figure 14:**
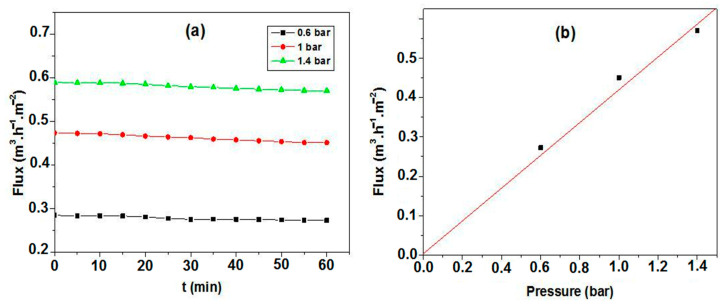
(**a**) Distilled water flux versus time, at different working pressures, (**b**) Water flux variation as a function of the different applied pressures for the membrane.

Distilled water was used to characterize the permeability at room temperature. The water flux through the membrane was measured as a function of time, at different transmembrane pressure (TMP) values. Figure 14 illustrates the obtained results. It is possible to observe that the increases in flux are related to changes in the pressure [52] with a stable flux that was reached after a few minutes. Moreover, a flux rate of 270 lh^−1^m^−2^ at 0.6 bar pressure, which increases to 580 lh^−1^m^−2^ at 1.4 bar, was also measured. The flux rate was found to increase with the increase in TMP. The permeability was estimated from the different flux values for each working pressure. Figure 14b presents the flux as a function of the applied pressures. It is observed that the flux increases linearly with the pressure; more precisely, the flux-versus-pressure curve follows the Darcy law. The linear relationship between the flux and the transmembrane pressure indicates that the driving force for fluid permeation is the pressure difference [52,53]. The obtained curve is a straight line with a slope equal to about 440; the permeability is estimated to be around 440 lh^−1^m^−2^bar^−1^,as can be seen in Figure 14b.

### 3.4. Efficient Sterilization by Membrane Filtration

**Figure 15:**
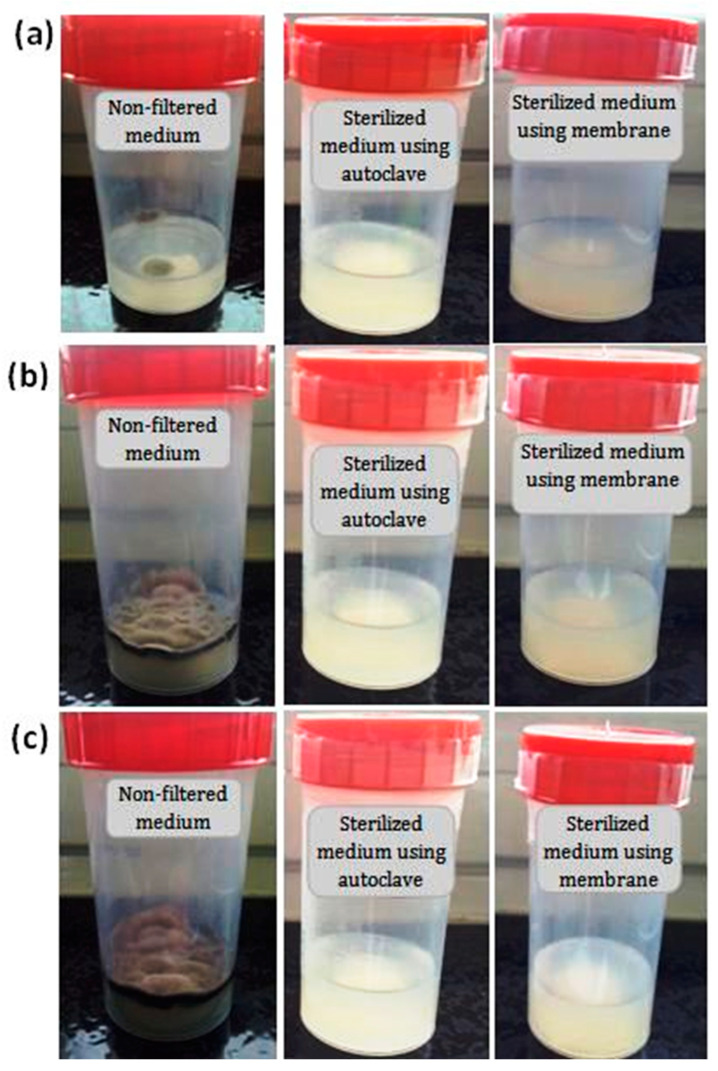
Photographs showing medium contamination variations versus time: (**a**) 5 days, (**b**) 15 days and (**c**) 30 days.

The filtration performance of a medium by porous zircon membranes was evaluated by following any changes to the medium over time. Figure 15 presents the photographs of the non-filtered medium, the filtered medium using zircon ceramic membranes, and the sterilized medium using autoclaving. According to this figure, it is possible to notice significant effects of the membrane on the sterilization process. It is clear that significant differences exist between the filtered and non-filtered media. Some variations in color were observed on the surface of non-filtered medium after five days of incubation. The presence of black and white spots is due to the bacterial growth; the development of a filamentous structure is an indicator of the presence of fungi mycelium. No microbial growth was detected on the surface of the filtered medium after one month of incubation. These results indicate that the developed membrane can entirely remove the microorganisms from the culture medium. Figure 15 shows also that the medium is satisfactorily clean. Therefore, this membrane proved to be efficient for the elimination of bacterial contamination. Studies on medium sterilization using zircon ceramic membranes are rare in the published literature. Compared to the conventional standard sterilization methods such as heat (autoclaving), the results of the present study indicate that it is possible to obtain a similar quality medium, achievable by a single step via a zircon-based membrane filtration process. The bacteria removal results of the prepared membrane are comparable to those of membranes prepared from clays and zirconia [53,54,55].

## 4. Conclusions

This work focused on the preparation and characterization of zircon porous ceramic membranes for application in microbial sterilization processes. They consist of a macroporous support system with an intermediate layer made of SS and a zircon microporous top layer. Membrane supports in the form of single-channel tubular and flat discs were made using SS (75 wt.%) and CC (25 wt.%) minerals. Flexural strength was found to depend strongly on the APS. Moreover, porous ceramic supports with the smallest APS exhibited the highest flexural strength. The porosity values ranged from 44 to 53%, the average pore size from 3 μm to 27 μm, the mechanical strength between 12 MPa and 20 MPa, and permeability was excellent, making them suitable for MF processes. Samples sintered at 1250 °C resulted in the best characteristics. Standard values of porosity, flexural strength, and permeability of SS-based membrane supports sintered at 1250 °C are estimated to be 45%, 15 MPa, and 95 m^3^h^−1^m^−2^bar^−1^, respectively.

The zircon deposited membranes prepared by the slip casting method showed the following characteristics:

- Average zircon layer thickness: 70 μm;
- Average pore size: 0.3 μm (suitable for MF application);
- Porous volume: 43%;
- Water permeability: 440 lh^−1^m^−2^bar^−1^;
- Good stability in aqueous solutions.

The efficiency of these membranes for bacteria removal was tested. Indeed, the obtained results are very encouraging given the high rate (100%) of retentions found experimentally.

Furthermore, bacteria removal performance results confirm the efficiency of zircon membranes for microfiltration, particularly for solutions sterilization; they are an alternative sterilization method to autoclaving.

## Figures and Tables

**Table 1:** Chemical compositions of raw materials, expressed as weight percentage, for the different oxides (obtained with the X-ray fluorescence technique).

	SiO_2_	CaO	Al_2_O_3_	K_2_O	SO_3_	MgO	ZrO_2_	Fe_2_O_3_	Na_2_O	TiO_2_
Silica sand	85.17	2.50	6.04	0.31	0.06	0.63	-	1.69	0.05	0.03
Calcite	0.06	55.95	0.06	0.01	0.04	0.33	-	0.02	0.06	-
Zircon	34.00	-	-	-	-	-	65.5	0.12	-	0.20

## Data Availability

Not applicable.

## References

[1] Li W., Ling G.Q., Huang P., Li K., Lu H.Q., Hang F.X., Zhang Y., Xie C.F., Lu D.J., Li H. (2016). Performance of ceramic microfiltration membranes for treating carbonated and filtered remelt syrup in sugar refinery. J. Food Eng..

[2] Ravanchi M.T., Kaghazchi T., Kargari A. (2009). Application of membrane separation processes in petrochemical industry: A review. Desalination.

[3] Iulianelli A., Drioli E. (2020). Membrane engineering: Latest advancements in gas separation and pre-treatment processes, petrochemical industry and refinery, and future perspectives in emerging applications. Fuel Process. Technol..

[4] Chen D., Sirkar K.K., Jin C., Singh D., Pfeffer R. (2017). Membrane-Based Technologies in the Pharmaceutical Industry and Continuous Production of Polymer-Coated Crystals/Particles. Curr. Pharm. Des..

[5] Goh K.S., Chen Y., Ng D.Y.F., Chew J.W., Wang R. (2022). Organic solvent forward osmosis membranes for pharmaceutical concentration. J. Membr. Sci..

[6] Yang C., Zhang G., Xu N., Shi J. (1998). Preparation and application in oil–water separation of ZrO_2_/α-Al_2_O_3_ MF membrane. J. Membr. Sci..

[7] Kanagaraj P., Nagendran A., Rana D., Matsuura T., Neelakandan S. (2015). Separation of macromolecular proteins and rejection of toxic heavy metal ions by PEI/cSMM blend UF membranes. Int. J. Biol. Macromol..

[8] Zhang R., Ma H., Wang B. (2010). Removal of Chromium(VI) from Aqueous Solutions Using Polyaniline Doped with Sulfuric Acid. Ind. Eng. Chem. Res..

[9] Karthik R., Meenakshi S. (2014). Removal of hexavalent chromium ions using polyaniline/silica gel composite. J. Water Process Eng..

[10] Rojas G., Silva J., Flores J.A., Rodriguez A., Ly M., Maldonado H. (2005). Adsorption of chromium onto cross-linked chitosan. Sep. Purif. Technol..

[11] Chitrakar H., Arun M.I., Mahesh P., Ahmad F.I., Lau W. (2012). New CPS-PPEES blend membranes for CaCl_2_ and NaCl_2_ rejection. Membr. Water Treat..

[12] Boulkrinat A., Bouzerara F., Harabi A., Harrouche K., Stelitano S., Russo F., Galiano F., Figoli A. (2020). Synthesis and characterization of ultrafiltration ceramic membranes used in the separation of macromolecular proteins. J. Eur. Ceram. Soc..

[13] van der Laan H., van Halem D., Smeets P., Soppe A., Kroesbergen J., Wubbels G., Nederstigt J., Gensburger I., Heijman B. (2014). Bacteria and virus removal effectiveness of ceramic pot filters with different silver applications in a long term experiment. Water Res..

[14] Medjemem N., Harabi A., Bouzerara F., Foughali L., Boudaira B., Guechi A., Brihi N. (2016). Elaboration and characterization of low cost ceramics microfiltration membranes applied to the sterilization of plant tissue culture media. J. Taiwan Inst. Chem. Eng..

[15] Majewska-Nowak K.M. (2010). Application of ceramic membranes for the separation of dye particles. Desalination.

[16] Vetten M.A., Yah C.S., Singh T., Gulumian M. (2014). Challenges facing sterilization and depyrogenation of nanoparticles: Effects on structural stability and biomedical applications. Nanomed. Nanotechnol. Biol. Med..

[17] Hussain A., Ahmed I., Nazir H., Ullah I., Annarita L. (2012). Plant Tissue Culture: Current Status and Opportunities. Recent Advances in Plant In Vitro Culture.

[18] Thomas P., Prabhakara B.S., Pitchaimuthu M. (2006). Cleansing the long-term micropropagated triploid watermelon cultures from covert bacteria and field testing the plants for clonal fidelity and fertility during the 7–10 year period in vitro. Plant Cell Tissue Organ Cult..

[19] Georg E.F., Hall M.A., De Klerk G.-J. (2008). The Components of Plant Tissue Culture Media I: Macro- and Micro-Nutrients. Plant Propagation by Tissue Culture.

[20] Gago J., Pérez-Tornero O., Landín M., Burgos L., Gallego P.P. (2011). Improving knowledge of plant tissue culture and media formulation by neurofuzzy logic: A practical case of data mining using apricot databases. J. Plant Physiol..

[21] Odutayo O.I., Oso R.T., Akinyemi B.O., Amusa N.A. (2004). Microbial contaminants of cultured Hibiscus cannabinus and Telfaria occidentalis tissues. Afr. J. Biotechnol..

[22] Wang C.-Y., Wang F., Wang T., Yang X.-L., Bian Y.-R., Kengara F., Li Z.-B., Jiang X. (2011). Effects of Autoclaving and Mercuric Chloride Sterilization on PAHs Dissipation in a Two-Liquid-Phase Soil Slurry. Pedosphere.

[23] Shi Y., Xu L., Gong D., Lu J. (2010). Effects of sterilization treatments on the analysis of TOC in water samples. J. Environ. Sci..

[24] Zhang J., Davis T.A., Matthews M.A., Drews M.J., LaBerge M., An Y.H. (2006). Sterilization using high-pressure carbon dioxide. J. Supercrit. Fluids.

[25] Faria L.F.F., Di Luccio M., Nobrega R., Borges C.P. (2002). Developement and characterization of microfiltration Hollow—Fiber modules for sterilization of fermentation media, Braz. J. Chem. Eng..

[26] Cruz R.M.S., Vieira M.C., Silva C.L. (2008). Effect of heat and thermosonication treatments on watercress (Nasturtium officinale) vitamin C degradation kinetics. Innov. Food Sci. Emerg. Technol..

[27] Perrut M. (2012). Sterilization and virus inactivation by supercritical fluids. J. Supercrit. Fluids.

[28] Leitzen S., Vogel M., Steffens M., Zapf T., Müller C.E., Brandl M. (2021). Quantification of Degradation Products Formed during Heat Sterilization of Glucose Solutions by LC-MS/MS: Impact of Autoclaving Temperature and Duration on Degradation. Pharmaceuticals.

[29] Chen M., Heijman S.G., Luiten-Olieman M.W., Rietveld L.C. (2022). Oil-in-water emulsion separation: Fouling of alumina membranes with and without a silicon carbide deposition in constant flux filtration mode. Water Res..

[30] Wehling J., Köser J., Lindner P., Lüder C., Beutel S., Kroll S., Rezwan K. (2015). Silver nanoparticle-doped zirconia capillaries for enhanced bacterial filtration. Mater. Sci. Eng. C.

[31] Kouras N., Harabi A., Bouzerara F., Foughali L., Policicchio A., Stelitano S., Galiano F., Figoli A. (2017). Macro-porous ceramic supports for membranes prepared from quartz sand and calcite mixtures. J. Eur. Ceram. Soc..

[32] Malekshahi M., Sabbaghi S., Rasouli K. (2022). Preparation of α-alumina/γ-alumina/γ-alumina-titania ceramic composite membrane for chloride ion removal. Mater. Chem. Phys..

[33] Boulkrinat A., Bouzerara F. (2021). Elaboration of tubular titania microfiltration membranes for wastewater treatment. Desalination Water Treat..

[34] Bouzerara F., Boulanacer S., Harabi A. (2015). Shaping of microfiltration (MF) ZrO_2_ membranes using a centrifugal casting method. Ceram. Int..

[35] Liu C., Wang L., Ren W., Rong Z., Wang X., Wang J. (2007). Synthesis and characterization of a mesoporous silica (MCM-48) membrane on a large-pore α-Al2O3 ceramic tube. Microporous Mesoporous Mater..

[36] Fan P.M., Zhen K.F., Zan Z.Y., Chao Z., Jian Z., Yun J.Z. (2016). Preparation and development of porous ceramic membrane supports fabricated by extrusion technique. Chem. Eng. Trans..

[37] Almandoz M., Pagliero C., Ochoa N., Marchese J. (2015). Composite ceramic membranes from natural aluminosilicates for microfiltration applications. Ceram. Int..

[38] Issaoui M., Limousy L. (2019). Low-cost ceramic membranes: Synthesis, classifications, and applications. Comptes Rendus Chim..

[39] Fang J., Qin G., Wei W., Zhao X. (2011). Preparation and characterization of tubular supported ceramic microfiltration membranes from fly ash. Sep. Purif. Technol..

[40] Biesheuvel P., Breedveld V., Higler A.P., Verweij H. (2001). Graded membrane supports produced by centrifugal casting of a slightly polydisperse suspension. Chem. Eng. Sci..

[41] Nijmeijer A., Huiskes C., Sibelt N.G.M., Kruidhof H., Verweij H. (1998). Centrifugal casting of tubular mem-brane supports. Am. Ceram. Soc. Bull..

[42] Chen C.-H., Takita K., Ishiguro S., Honda S., Awaji H. (2005). Fabrication on porous alumina tube by centrifugal molding. J. Eur. Ceram. Soc..

[43] de la Rocha M.R., Virginie M., Khodakov A., Pollo L.D., Marcílio N.R., Tessaro I.C. (2021). Preparation of alumina based tubular asymmetric membranes incorporated with coal fly ash by centrifugal casting. Ceram. Int..

[44] Jedidi I., Saïdi S., Khmakem S., Larbot A., Elloumi-Ammar N., Fourati A., Charfi A., Ben Amar R. (2009). New ceramic microfiltration membranes from mineral coal fly ash. Arab. J. Chem..

[45] Khemakhem S., Ben Amar R., Ben Hassen R., Larbot A., Medhioub M., Ben Salah A., Cot L. (2004). New ceramic membranes for tangential waste-water filtration. Desalination.

[46] Masmoudi S., Ben Amar R., Larbot A., El Feki H., Salah A., Cot L. (2005). Elaboration of inorganic microfiltration membranes with hydroxyapatite applied to the treatment of wastewater from sea product industry. J. Membr. Sci..

[47] Bagdassarov N., Delépine N. (2004). α–β Inversion in quartz from low frequency electrical impedance spectroscopy. J. Phys. Chem. Solids.

[48] Kadiri C., Harabi A., Bouzerara F., Foughali L., Brihi N., Hallour S., Guechi A., Boudaira B. (2020). Preparation and properties of tubular macroporous ceramic membrane supports based on natural quartz sand and dolomite. J. Aust. Ceram. Soc..

[49] Zhou J., Zhang X., Wang Y., Larbot A., Hu X. (2010). Elaboration and characterization of tubular macroporous ceramic support for membranes from kaolin and dolomite. J. Porous Mater..

[50] Kaur H., Bulasara V.K., Gupta R.K. (2016). Effect of carbonates composition on the permeation characteristics of low-cost ceramic membrane supports. J. Ind. Eng. Chem..

[51] Rouquerol J., Avnir D., Fairbridge C.W., Everett D.H., Haynes J.M., Pernicone N., Ramsay J.D.F., Sing K.S.W., Unger K.K. (1994). International Union of Pure and Applied Chemistry Physical Chemistry Division Commission on Colloid and Surface Chemistry, Subcommittee on Characterization of Porous Solids: “Recommendations for the characterization of porous solids (Technical Report)”. Pure Appl. Chem..

[52] Bazin M.M., Ahmad N., Nakamura Y. (2019). Preparation of porous ceramic membranes from Sayong ball clay. J. Asian Ceram. Soc..

[53] Vasanth D., Pugazhenthi G., Uppaluri R. (2011). Fabrication and properties of low cost ceramic microfiltration membranes for separation of oil and bacteria from its solution. J. Membr. Sci..

[54] Kumar C.M., Roshni M., Vasanth D. (2019). Treatment of aqueous bacterial solution using ceramic membrane prepared from cheaper clays: A detailed investigation of fouling and cleaning. J. Water Process Eng..

[55] Kroll S., Treccani L., Rezwan K., Grathwohl G. (2010). Development and characterisation of functionalised ceramic microtubes for bacteria filtration. J. Membr. Sci..

